# WOMEN AND WORK AT MID LIFE AND BEYOND: STUMBLING, STRIVING, NAVIGATING TRANSITIONS

**DOI:** 10.1093/geroni/igad104.1918

**Published:** 2023-12-21

**Authors:** Julie Miller

## Abstract

Women at midlife and beyond are making significant and increasing contributions to The Global Longevity Economy, driving increases in Gross Domestic Product (GDP), job creation, and income generation. Indeed, advances in women’s life expectancies, statuses, and roles establish women aged 50-plus as a high-powered engine driving economic opportunity. As much as we can and must celebrate the economic contributions of older women, we cannot do so without acknowledging the forces holding them back from contributing even more. Despite the progress women at midlife and beyond have made in the United States relative to many of those who came before them, they still are challenged to reach their ultimate economic potential. This session will explore some of the factors that propel and inhibit women aged 50-plus from reaching their full economic potential, each of which is related in some capacity to paid work. The first presentation in this symposium spotlights a mixed methods study into the economic lives of working women at midlife and beyond across the United States who face one or more barriers, in addition to gender, to building their economic security. The second presentation will focus on financial challenges and experiences of women entrepreneurs aged 40-plus during the Covid-19 pandemic. The final presentation will explore financial and professional impacts of menopause transitions for women. Taken together, implications across all studies point to broad areas of intervention, innovation, and additional areas for scholarship – all as they relate to financial wellbeing and work for women at midlife and beyond.

